# Exploring the barriers and enablers of oral health care utilisation and safe oral sex practices among transgender women in Malaysia: a qualitative study

**DOI:** 10.1186/s12889-025-22417-9

**Published:** 2025-04-03

**Authors:** Lahari A. Telang, Hezreen Shaik Daud, Abdul Rashid, Aoife G. Cotter

**Affiliations:** 1 School of Medicine, University College Dublin, Dublin, Ireland; 2 Penang International Dental College, Penang, Malaysia; 3 RCSI-UCD Medical Campus (RUMC), Penang, Malaysia; 4 Penang Family Health Development Association (FHDA), Penang, Malaysia; 5 UCD Centre for Experimental Pathogen Host Research (CEPHR), Dublin, Ireland; 6 Mater Misericordiae University Hospital, Dublin, Ireland

**Keywords:** Transgender women, Malaysia, Oral health needs, Dental service utilisation, Oral transmission of STIs

## Abstract

**Background:**

Transgender women in Malaysia face social and healthcare marginalisation. Research about their oral health and oral health care utilisation is sparse. Despite growing clinical evidence highlighting the risk of transmission of sexually transmitted infections (STIs) through oral sexual practices, research in this area remains less explored. This study aimed to understand the experiences of transgender women in Malaysia by exploring oral health care needs and the barriers and enablers of oral health care utilisation as well as safe sexual practices relating to oral transmission of STIs.

**Methods:**

Participants were recruited through a snow-balling method of sampling with the help of community workers. Semi-structured in-depth interviews (IDIs) with transgender women in northern Malaysia and Focus group discussion (FGD) with a mixed group of transgender women and health care professionals were conducted to gain insights into the needs of the community. Data obtained from IDIs and FGD were coded, transcribed, and thematically analysed to derive codes and themes through the interpretative lens of the Information, Motivation and Behavioural skills (IMB) theory.

**Results:**

Participants of the IDIs were transgender women (*n* = 20, median age 39.8 (9.75 IQR) years). Aesthetic dental needs were prioritised, yet poor utilisation of dental services was reported, with many opting for self-medication or care from a non-qualified dental practitioner. Routine engagement in oral sex practices, primarily receptive fellatio with or without ejaculation with multiple cis-gender male partners, was reported. Low awareness of oral STIs, along with a perceived low risk of transmission of STIs through oral sex, was reported, with most (18, 90%) not using condoms for clients/partners or inconsistently using them during oral sexual practices. The themes identified from IDIs and FGD included: *‘Place in the society’* ‘*Attitudes and beliefs linked with dental care’*,* ‘Access to dental care’*,* ‘Lack of trans-specific health care’* and ‘*Use of condoms for oral sex’*.

**Conclusion:**

The study’s findings report poor dental service utilisation among transgender women despite aesthetics being prioritised. Gaps in knowledge regarding the oral transmission of STIs were also noted. These insights underscore the need for trans-specific health campaigns designed to address these concerns and enhance awareness through an integrated approach to improve access to inclusive oral health care and sexual health care for this vulnerable population.

**Supplementary Information:**

The online version contains supplementary material available at 10.1186/s12889-025-22417-9.

## Background

Transgender women are individuals who were assigned male at birth but identify and live as female [1–3]. In Malaysia, transgender women are referred to as *“mak nyahs”*, which was once considered to be a respectful term to describe effeminate, lady-like men [4, 5]. However, today, they face stigma and discrimination and the threat of criminal prosecution by both the civil and religious laws of the country [5, 6]. Additionally, media reports often use incorrect or demeaning labels when referring to transgender individuals, further reinforcing harmful misconceptions [7]. The inability to express their identities freely leads to them being marginalised by society and/or choice. Such marginalisation may push them to sex work as a means of earning a living [6, 8, 9]. A confluence of structural, environmental, social, and individual factors drive heightened sexual risk behaviours among transgender women, such as sex work and unprotected sexual activities [2, 10]. Health and well-being are negatively impacted as the basic determinants of health, and social security, education, employment, family support, and health care are undermined by stigma and legal processes [11, 12].

The Ministry of Health, Malaysia recognises transgender women as one of the key population groups who are vulnerable and have a high prevalence of Human immunodeficiency virus infections (HIV) and sexually transmitted infections (STIs) based on the National Strategic Plan for Ending AIDS report (NSPEA 2016–2030) [2, 13]. The estimate of transgender women living with HIV was reported to be 10.9% in 2017 [14]. However, among transgender women sex workers, higher estimates of those living with HIV were reported at up to 12.4% in 2018, alongside high rates of syphilis [10]. The emphasis on prevention of HIV/STIs has historically been focused on protection during anal sex for both men who have sex with men (MSM) and transgender women [15, 16]. Even when it is well recognised that transmission of STIs, both viral and bacterial, can occur through oro-genital and oro-anal routes, less emphasis is placed on these routes of transmission in preventive programs [16, 17]. This may be because STIs in the oral and oro-pharyngeal regions are mostly asymptomatic, with manifestations seen less often in the anterior part of the oral cavity and causing only an occasional sore throat when harboured in the tonsil or pharynx [16, 18]. Growing concerns about transmission of human papillomavirus (HPV) through oral sexual contact and HPV-related oropharyngeal cancers have also been heightened in recent years [19, 20]. Moreover, the oral cavity’s health can directly impact the transmission of infections. The oral cavity’s microbial environment and potential for trauma make it a gateway for pathogens [20, 21]. Unhealthy periodontal conditions, open sores or ulcers of the oral mucosa and bleeding from the gums are known to increase the risk of transmission of STIs [21]. Reports regarding oral sexual practices and behaviour, along with the use of protection for oral sex among transgender women in Malaysia, are lacking. Hence this route of transmission remains less explored.

Malaysia is an upper-middle-income country and one of the few Asian countries that has achieved Universal Healthcare Utilisation (UHC) [22]. This means that access to health care services, including sexual, mental, and oral health, is ensured to all citizens without exposing the user to financial hardship [23, 24]. Although healthcare is accessible for all, it fails to address the multiple healthcare needs of transgender women [11]. This could be because of the poor cultural acceptance of transgender persons by the community, including health care providers and also by the transgender woman herself, who feels she is stigmatised and is reluctant to attend services for fear of experiencing discrimination, thus creating barriers to rightful utilisation of health care services [11, 25]. 

Utilisation of oral health care in Malaysia is reported to be generally low across all population groups despite the availability of care, thus leading to a higher burden of oral diseases [26]. According to the World Health Organization (WHO), a disproportionately higher burden of oral diseases affects the most vulnerable and disadvantaged populations of the world [27]. One report on transgender women in Malaysia suggested they have poorer oral health-related quality of life compared to the general adult population [28]. However, studies on oral health needs and oral health care utilisation of vulnerable populations are sparse in the developing parts of the world [29]. The emphasis on oral health care utilisation to facilitate early detection of oral STIs and creating awareness regarding oral transmission of STIs also deserves attention. The study employs an exploratory approach to identify the oral health care needs of transgender women in Malaysia and to understand their experiences of utilisation of oral health services. Additionally, the behavioural factors that act as both barriers and enablers to safe sexual practices related to the oral transmission of STIs have been examined.

## Methodology

This study was conducted in Penang, a northern state of Malaysia with a multi-ethnic and multicultural society. Penang has the highest population density of all Malaysian states and a high rate of urbanisation [30]. This study was undertaken as a preliminary part of a larger study examining oral and sexual health related to oral STIs in transgender women in Malaysia. Varied purposive sampling methods were used, through community organisations based on predefined criteria, as well as volunteer response sampling via text messages [31]. A qualitative analysis methodology was adopted to gain an in-depth understanding to uncover the nuances and provide meaning to this exploratory research. The sample size for the interviews was based on saturation in data, determined as the point after which no new information was obtained, and any further data collection through subsequent interviews would be redundant [32]. A participatory approach for designing the study was adopted, with transgender women from the local community involved in planning. Initial meetings between the principal author (LAT) and a few transgender women community advocates were conducted in informal settings to explore the general frame of mind of the community. Community advocates provided feedback regarding the study design and assisted with recruiting participants. They not only supported the interpretation of the preliminary data but also the follow-up part of the study. The Health Belief Model (HBM) [33] and Andersen’s Behaviour Model of Health Services Use [34] were used as frameworks to devise the open-ended questions guide for the semi-structured interviews (Supplementary file 1) and focus group discussion.

### Ethics

The study was approved by the University College Dublin, UCD Human Research Ethics Committee – Sciences (HREC-LS) (LS-C-22-158-Telang-Cotter), and the Joint Penang Independent Ethics Committee (JPEC 22 − 0002). The participant information sheet and informed consent were made available in English and translated into the local language (Bahasa Malaysia).

### In-depth interviews

The initial participants were referred by a local community-based welfare organisation working with transgender women. Subsequent participants were recruited through word of mouth using a snow-balling sampling method and belonged to several friendship networks. Invitations to participate were sent via text messages from the author (HSD) to potential participants. Any individual identifying as a transgender woman, able to give informed consent, aged 18 years and above, was invited to participate in the study. Written informed consent was obtained, and semi-structured in-depth interviews were conducted. Face-to-face interviews were conducted with most participants, with only two interviews conducted as group interviews with two participants at each interview. This was done to accommodate participants’ personal choices and preferences. All interviews were held in a safe space based on the participants’ convenience and audio-recorded using an audio recorder. The interview guide was developed by the authors based on prior research evidence in local transgender health [6, 35] and was guided by community advocates who were involved in healthcare outreach. The interview questions were flexibly used to initiate the conversation and to understand the participants’ views. (Supplementary file 1). The interview questions revolved around broad themes of demographics, their place in the community and social support, oral health care and visiting the dentist, sexual health and practices, and oral sex and protection. The participants were compensated for their time and travel expenses (approximately USD 12). The interviews lasted for 30 to 90 min in duration. They were conducted by both the principal author (LAT), a cis-gender woman, and a trained research assistant (HSD), who is a transgender woman with experience in transgender health outreach and advocacy. English and Bahasa Malaysia were both used during the interviews, with facilitation by the research assistant to better understand variations in local dialects and appropriate cultural nuances. Most participants were able to communicate effectively in both English and Bahasa Malaysia. A combination of direct translation and a back-and-forth translation of data was employed, with certain nuanced statements preserved in their original form. The research team, comprised of native Bahasa Malaysia language speakers as well as a native English language speaker, conducted multiple reviews of the data to ensure clarity and coherence in both languages. Documentation of non-verbal cues during the interview was conducted using handwritten notes and references were drawn after the interview.

### Focus group discussion

The preliminary data from the interviews revealed key insights into the needs of the community and broad perspectives on the barriers and facilitators of oral and sexual health care. Multiple stakeholder feedbacks were deemed essential to obtain a more detailed understanding of the situation. This was important for consolidating and validating the themes derived from the interviews. Hence, a focus group discussion with diverse participants was conducted. Participants were recruited through purposeful sampling by including members who would contribute significantly to the discussion and were directly or indirectly involved with transgender women’s health in Malaysia. The discussion lasted over two and a half hours, with each participant validating the concerns raised through the codes generated from the interviews. The discussion also contributed information regarding the current position of transgender women in society, safe sexual practices, and healthcare utilisation.

## Coding and data analysis

Interview data were transcribed and coded using the NVivo 12 Windows software. Subsequently, a multistep coding process was performed. The initial auto-generated codes were compared with those manually generated using the software, and the investigators reached a consensus. The most important ideas that emerged were coded, and similar codes were clustered into minor themes. Thematic analysis was used to interpret and derive major themes [36]. Direct quotations from participants about their experiences, opinions, knowledge, and feelings were also recorded. The methodological principles of Grounded Theory and Narrative inquiry guided the analysis [37]. A similar data coding and analysis process was adopted for the focus group discussion data.

### Patient and public involvement

Public members from the transgender community in Penang were involved in initial discussions regarding the study settings. Their opinions regarding safe locations for conducting the interviews and using culturally appropriate language were considered while designing the interview guide. Participant recruitment was done with the help of community advocates. One of the authors is a transgender woman from the local community with experience in transgender health outreach and advocacy. All interviews were conducted by her, along with the principal author. She has also contributed to interpreting interview data and translation and consultations with transgender community advocates.

## Results

Demographic data of the interview participants was collected, including socio-economic data, sexual, medical, and dental history, as outlined in Table 1. Participants in the in-depth interviews identified as transgender women (*n* = 20), with all having a heterosexual/straight sexual orientation and sexually (physically and emotionally) attracted to men. The median age of the participants was 39.8 (9.75 IQR) years. Participants lived in urban communities, most hailing from Penang (*n* = 16), three from the neighbouring state of Kedah, and one from Perak. The majority had a secondary school level of education (*n* = 16(80%)), with a few receiving tertiary education (*n* = 4 (20%)). The majority were ethnic Malay Muslims, 18 (90%), with one participant belonging to the Chinese ethnicity and one ethnic Indian Muslim.

Six (30%) of the 20 participants who were interviewed engaged in sex work for a living and five (25%) participants were involved in part-time sex work. All participants were aware of HIV, its prevention, and the availability of treatment in public healthcare settings. Twelve (60%) participants had been tested for HIV in the last 6 months and were negative at the last test; six (30%) were unaware of their status, and two (10%) were currently on pre-exposure prophylaxis (PrEP). There was a general awareness of the term *“Jangikat kelamin”* (in Bahasa Malaysia), which translates to sexually transmitted infections. Still, awareness of types of non-HIV STIs, differential risks and treatments was low. When names of common STIs (syphilis, gonorrhoea, chlamydia, herpes) were listed in the local language, most participants demonstrated either verbal or non-verbal cues of unawareness. Participants who were full-time sex workers had relatively better awareness, with four (20%) of the six having heard of syphilis before the interview.

All participants routinely engaged in sexual intimacy with clients or partners through oral sexual practices (20(100%)) and often with multiple partners (12(60%)). In this context, oral sex was primarily receptive fellatio (oro-genital contact) with or without ejaculation with cis-gender male partners, which may or may not be accompanied by anilingus (oro-anal contact) on male partners. Participants perceived no risk of HIV transmission (20(100%)) through oral sex and a low risk of transmission of STIs (16(80%)). Most (18(90%)) never used condoms/barriers or inconsistently used them for their partners during receptive fellatio, with or without ejaculation.

Routine oral hygiene using tooth brushing with toothpaste was practised by all participants, with only a few using additional aids such as mouthwash (5 (25%)) or flossing (2 (10%)) of teeth. All the participants were aware of the importance of oral health yet reported low utilisation of dental services, with the majority (16(80%)) not having visited a dentist since school days. Smoking and vaping with electronic cigarettes was common practice with all the participants, along with occasional substance/ drug misuse by six (30%) (e.g., methamphetamine).

**Table 1:** Demographic characteristics of in-depth interview participants

Participant characteristics	*n = 20 (100%)*
*Demographics*:	
Lifestyle- Urban Living	20 (100%)
Ethnicity	
Malay	18 (90%)
Chinese	1 (5%)
Indian	1 (5%)
Education	
Completed Secondary school	16 (80%)
Completed Tertiary level education	4 (20%)
*Sexual History*:	
Transactional sex/sex work	
Full time	6 (30%)
Part-time	5 (25%)
None	9 (45%)
Sexual partners in the past 1 month	
Single	8 (40%)
More than one	12 (60%)
HIV and STI current status (testing in last 6 months):	
Unaware of status	6 (30%)
Aware and negative	12 (60%)
Aware and taking PrEP	2 (10%)
Engagement in with client/partner oral sex practices in the past month (oro-genital and/or oro-anal)	
Regular	20(100%)
Condom usage with client/partner during oral sex practices in the past month (oro-genital and/or oro-anal)	
Always	2 (10%)
Sometimes	3 (15%)
Never	15 (75%)
Perceived risk of transmission of STIs during Oral sex (through oro-genital and/or oro-anal route)	
Moderate-high risk	4 (20%)
Low risk	16 (80%)
*Dental History*:	
Oral hygiene practices	
Brushing with toothpaste	20 (100%)
Mouthwash use	5 (25%)
Flossing	2 (10%)
Frequency of dental visits in a 12-month period	
Sometimes	4 (20%)
Never	12(60%)
Never, do-it-yourself/ non-qualified dental practitioner/ beautician (Fake dentures/veneers)	4 (20%)
Smoking cigarette	20(100%)
Vaping (E-cigarettes)	20(100%)
Substance/ drug misuse, e.g.,* Meth (methamphetamine)*	
Never	14 (70%)
Occasional	6 (30%)

(HIV: Human immunodeficiency virus, STIs: sexually transmitted infections, PrEP: Pre-exposure prophylaxis, E-cigarettes: Electronic cigarettes)

Participants of the FGD (*n* = 7) included four transgender women, two of whom were local community advocates, one activist, and one artist. The other three participants were cis-identified and included a public health physician from the local STI “friendly clinic,” a psychological counsellor specialising in gender and sexual minorities’ matters, and a general dentist who was working for a local non-governmental organisation (NGO) for women’s welfare and vulnerable groups.

### Themes

Data from the interviews and FGD was interpreted using a combination of inductive and deductive approaches. Results were represented as participants’ perspectives and researchers’ interpretations. While the in-depth interviews ascertained the participants’ personal beliefs and perspectives, the FGD provided shared views of the interactions between the participants. Integrating both data sources under the same themes provided a more comprehensive overview. The data analysis was guided by the interpretative lens of the Information, Motivation and Behavioural Skills (IMB) theory [38]. The major themes were *‘Place in the society’*, ‘*Attitudes and beliefs linked with dental care’*,* ‘Access to dental care’*,* ‘Lack of trans-specific health care’* and ‘*Use of condoms for oral sex’*. The findings from the thematic analysis is summarised in Table 2.

**Table 2:** Summary of findings from thematic analysis of results

Themes	Codes	
	Barriers	Enablers
Place in the society	- Stigma and discrimination - Lack of equal opportunities - Feeling targeted as they stand out - Religious and political influences contribute to marginalisation	- Friendship networks and sisterhood - Community workers and NGOs
Attitudes and beliefs linked with dental care	- Perceived lack of need for dental care - Fear and anxiety - Association with pain	- Beautiful smile attracts clients - Importance of teeth for a feminine appearance
Access to dental care	- Opening hours and long waiting times at public healthcare facilities - Poor public transportation - Prohibitive cost of private dental care - Lack of self-confidence to face the dentist	- Free treatment at public health care facilities - Easy access to alternatives (Online self-care remedies) - Quick fixes by beauticians/ fake dentists
Lack of trans-specific healthcare	- Fear of discrimination and feeling targeted - Fear of diagnosis - Lack of trained doctors and staff - Lack of confidence in the ability of the doctor - Less “friendly clinics” in rural areas - Dislike being subsumed with MSMs in the same clinics	- “Friendly clinics”- public health care facilities - Urban living - NGOs and community welfare - Support through a transgender network
Use of condoms for oral sex	- Perceived low risk of oral sex - Lack of awareness of oral manifestations of STIs and transmission - Partner preferences - Taste of condoms - Limited supply of condoms	- Education and awareness - Full-time sex workers

### Place in the society

Participants were generally unhappy about their place in society as they felt they were being treated as second-class citizens in their own country and let down by their own people. Lack of job opportunities was attributed to their appearance, which was different from the more normalised cis-gender appearance. Moreover, the existing religious and political environment was an influencing factor that contributed to marginalisation.

*“I want them (referring to cis-gendered persons) to see that even though I am a transwoman*,* I can do my job properly*,* our community is good*,* we can do the same jobs*,* don’t look at me differently!” -Respondent 013IDI a transgender woman.*

*“I feel transwomen are targeted because we are obvious and stand out in the crowd. The others (referring to lesbian*,* gay*,* and bisexual individuals)… L*,* G*,* and B can hide. But the “T” (transgender individuals)… we cannot hide! The religious leaders spread hatred towards us.”-Respondent 003FDG*,* a transgender woman.*

### Attitudes and beliefs linked with dental care

Feminine appearance was invariably seen as a crucial element during gender affirmation, and the importance of a beautiful smile was recognised as a criterion for attracting clients for sex work. More interest was expressed for aesthetic procedures such as dental veneers and not much for general oral health.

*“Yes*,* looks are very important for my girls… the pretty ones are in demand…your smile is important.”- Respondent 007IDI*,* a transgender woman sex worker.*

*“Healthy gums and teeth are pre-requisites for dental veneers*,* it is not a quick fix job… many patients don’t understand that… that is why they go to quacks (referring to non-professional treatment offered by beauticians).”- Respondent 007FGD*,* a cis-identified general dentist.*

However, Oral health was considered low in priority, with most participants not having been for a dental visit since school days. The visit to a dentist was associated with pain, fear, and anxiety, making it seem necessary only when a genuine problem such as toothache arises.

*“I am scared to go to the dentist; I don’t like noise and the smell.”- Respondent 0012IDI*,* a transgender woman.*

*“I think it is enough to go to the dentist when you have pain only.*”-*Respondent 006FDG*,* a transgender woman community worker.*

### Access to dental care

Participants were all aware that the public health care dental clinics provided free of cost treatment for all basic dental needs. Although discrimination per se in the public dental setting was not a concern that was highlighted, discontent with long waiting hours, unsuitable clinic operating hours and lack of public transport to reach the clinic were quoted as reasons for not utilising them. Some preferred private dental setups, citing confidentiality concerns and an overall lack of confidence in the care quality.

*“Oh (exclaims) the government clinic (public dental clinic)… They take so long… Sometimes I wonder if they know what they are doing”. “I prefer the private dental clinic in my neighbourhood…I can go in the evenings… I cannot get up early and line up… I work at night” -Respondent 019IDI*,* a transgender woman sex worker.*

One of the participants cited a preference for unbiased and cheaper dental care provided in the neighbouring country, Thailand.

*“I prefer to go to Bangkok for dental; the veneers are cheaper. Also*,* they don’t question you about what shade you want. I like mine as white as possible*,*… glow in the dark kind (says it with a quirky smile).”-Respondent 0018IDI*,* a transgender woman sex worker.*

A few participants considered self-care remedies from the internet and cheaper and quicker fixes provided by fake dentists or beauticians acceptable. One participant had even fixed her tooth herself.

*“I got this online and fixed it myself (showing off her plastic incisor)”. “It is very easy to buy fake braces or fake veneers online… very cheap also.”-Respondent 001IDI*,* a transgender woman.*

*“They (referring to beauticians) can do everything in one sitting at your home… spa*,* facial*,* cleaning of teeth and even teeth whitening.” Respondent 004IDI*,* a transgender woman.*

### Lack of trans-specific healthcare

The participants, in general, were of the opinion that healthcare facilities in Malaysia were available but not very accessible to transgender women. Inaccessibility was pinned on discrimination at healthcare facilities, mainly by the staff and less often by the doctors themselves.

*“I am paying taxes… I can use the clinic and hospital facility for free just like anybody else… I have been asked*,* “How long am I planning to act like a woman?” on my face…I get angry just to think of that. How can the hospital staff talk to me so rudely…? I don’t want to go back to that place ever again… (Emotional and teary)- Respondent 011IDI transgender woman*.

Another common concern was the lack of availability of gender affirming care or even the familiarity with hormone therapy by healthcare providers. This deters transgender women from seeking healthcare advice, especially during their gender affirmation phase. The participants pointed out that they lacked confidence in the ability of the doctors to manage trans-specific health concerns.

*“It is very easy to buy hormones from the pharmacy”*,* but if you must go through the proper channel*, they will send you to see a shrink (psychiatrist)… to see if you really are a transgender in your head… (rolls her eyes as she speaks)… only then the doctor can prescribe you the hormones… How many of us can go through that? ***… (swears)… so we rely on each other and online information. - Respondent 005IDI,* a transgender woman.*

The Ministry of Health’s ‘friendly clinics” were set up a few years ago that cater specifically to the HIV/STI needs of high-risk key populations. Still, there are very few such clinics available mostly in urban areas. Limited training provided to the doctors and staff was also cited as a concern.

*“Only minimal training regarding transgender-specific health is given to the doctors who manage the “STI-friendly clinics”*,* a one-day workshop or so…it is up to the individual doctor to build a rapport and convince the patients to come for regular follow up.”- Respondent 002FGD*,* a cis-identified public health physician.*

Transgender women in the study were all residing in urban areas. They believed they were better aware of HIV/STIs because of access to awareness campaigns compared to those living in rural areas. The support network provided by the transgender community was seen as an important aspect during the transition journey of every transgender woman. This support was stated to be stronger in urban communities.

*“Being a transgender woman and being Muslim is difficult… The struggle is real*,* especially in rural areas. That is why they live in big cities for better community support and jobs.”- Respondent 001FGD*,* a transgender woman community worker.*

The Ministry of Health conducts HIV/STI health campaigns with a focus on anal and genital sex. However, participants could not recall information from these campaigns about other types of STIs, and even less about the oral transmission of STIs.

*“I have attended health talks so many times. No one told me about oral STIs… (looks surprised)… Can you get STIs in the mouth also? I did not hear about that.”- Respondent 020IDI*,* a transgender woman.*

Many of the participants perceived such campaigns to be overloaded with information with little, if not any, new message to take home. Transgender women are often subsumed with MSMs and other key population groups in these campaigns, making them feel targeted.

*“I don’t like to be mixed with the MSMs and gays. We are different and look different… the KKM (government clinics) is always targeting us!” Respondent − 005IDI transgender woman sex worker.*

The NGOs play a crucial role as they work closely with community groups by employing members from key populations. They help spread awareness to transgender women in rural areas who may be difficult to reach.

*“It is difficult to reach the ones who default treatment (referring to HIV). We work closely with the NGOs*,* and the community is the one that lends a helping hand for their own kind”- Respondent 002FGD*,* a cis-identified public health physician.*

The transgender women community workers believed those who newly transitioned did not pay attention to any of these campaigns and preferred to lead a risky life. For some the fear of “getting diagnosed” with a disease prevented them from seeking medical advice or even testing for HIV/STIs. A general lack of information regarding PrEP was also noticed.

*“The NGOs do programs sometimes… even about health*,* law enforcement*,* but the transgender women*,* especially the young ones…don’t want to go*,* even they give some incentives also… no they don’t care… Maybe they think it’s not important.”-Respondent 003 FGD*,* a transgender woman community worker.*

### Use of condoms for oral sex

The participants were well aware of the transmission risks of HIV and STIs through the genital and anal routes. Even though awareness of other types of STIs and their differential risks was not there, the general perception of transmission of STIs through oral sex was considered negligible or very low. Many of the transgender women having casual sex partners did not seem to think of oral sex (oro-genital and/or oro-anal) as a potential source of the spread of infection. The only concern was ejaculation in the oral cavity, for which the opinion was washing the mouth after the act would be sufficient to keep it clean.

*“Saliva is antiseptic”… You don’t need condoms for oral sex! (Looks surprised)…-Respondent 020IDI*,* a transgender woman.*

General awareness regarding oral manifestations of STIs was also low, with none of the participants able to recall any oral lesions because of oral sex other than occasional trauma to the mucosa.

*“Ulcers… I don’t know*,* but when I sometimes involve in heavy oral sex*,* I have injuries in the mouth”-Respondent 008IDI*,* transgender woman.*

The lack of condom usage for oral sex with a client or partner was attributed to the unpleasant taste of the condom itself and the limited supply of condoms, which they would rather reserve for anal sex.

*“We get a limited supply of free condoms from the NGO. The free ones are the cheap ones*,* no flavour… don’t taste good…(frowns)” -Respondent 019IDI*,* a transgender woman sex worker.*

Condom use was found to differ with partner type. Especially for receptive fellatio with or without ejaculation, condoms were not used for regular trusted partners or for clients who paid more and insisted on not using condoms.

*“I don’t have a partner*,* but I have many friends who are boys… friends with benefits” “I am aware*,* (says reluctantly)… I know condoms are needed for a blowjob” (receptive fellatio)…but… “If I know the guy is very clean… I don’t ask him to wear a condom… Maybe one person out of ten wears a condom…. But…when you fall in love*,* you don’t think of condoms”-Respondent 002IDI*,* a transgender woman.*

## Discussion

The results of the qualitative data presented in the study examine two key aspects of oral health in transgender women: their needs and utilisation of oral health services and sexual health practices related to the oral transmission of STIs. These areas have been intentionally integrated to underscore the critical role of oral health care in identifying early signs of STIs, which are often overlooked, and to highlight the frequently neglected issue of STI transmission through unprotected oral sex [16, 39]. The interviews and focus group discussion detailed the broad assumptions regarding transgender women in Malaysia. Although the study primarily focused on oral health care and the oral transmission of STIs, discussions naturally extended to wider topics such as societal inclusion, living conditions, and overall health needs. These topics are integral to the daily lives of the local transgender women. The socio-demographics of the participants were similar to transgender women from other urban parts of Malaysia in terms of education, engagement in sex work and HIV/STI testing rates [2, 6, 40]. They reported experiencing marginalisation, being unable to express their identities and general discontent with their place in society, much similar to that identified by research from other urban transgender women in Malaysia [2, 6]. Lack of job opportunities, stigma and discrimination experienced at various fronts, including health care settings, were similar to those reported by transgender women belonging to other developing parts of the world, including Malaysia [9, 41, 42], as well as in a larger global context [42, 43]. 

### Oral health care needs and utilisation of dental services

This study was one of the first to explore oral health care needs and dental services utilisation of transgender women in this part of the world. The oral healthcare needs of the transgender women in this study were not found to be trans-specific. Oral hygiene practices and frequency of dental visits were also seen to be similar to that of the general adult population [26]. The participants believed that it was important to have an attractive smile in order to look feminine, as appearance was a key element to gender affirmation, which is supported by findings from similar studies [6]. Despite this, visiting a dentist was perceived as only needed when there was pain or a specific complaint. The participants associated dental treatment with fear and anxiety. The perceived lack of need for dental care and the avoidance of dental visits are complex issues deeply rooted in cultural beliefs and societal norms [44, 45]. Studies have linked the association of dental fear with experiences of discrimination and maltreatment and self-reported poor oral health [46–48]. 

Barriers to utilising free public dental health services were attributed to long waiting times, unsuitable opening hours, or the lack of transportation to reach the clinic. These barriers were identified to be more general and widespread in the community [26, 44] and not exclusive to transgender women. On the other hand, private dental care was deemed expensive and hence out of reach for many. This gap in the availability of suitable dental services may be a cause for turning to neighbouring countries or even fake dentists or unprofessional do-it-yourself dental treatments, which has become a growing concern in Malaysia [45, 49]. Fake dentists are beauticians who provide quick aesthetic dental fixes in the comfort of the client’s homes or hotel rooms without the need for long waiting [45, 50]. Since feminine appearance and looking attractive to a client or partner were given priority over the health of the teeth and gums, unprofessional aesthetic dental care may have been perceived as a more accessible option. The utilisation of oral health care services by transgender women in Malaysia may thus be influenced by socioeconomic and demographic factors much similar to the adult population in the country [26]. Influence of socio-economic factors on dental service utilisation by transgender populations has also been reported from other parts of the world [47, 51–53]. 

Critical factors that determined their decision to utilise public health services, including HIV/STI clinics, were influenced by the socio-political environment and fear of discrimination at health care centres, as was mentioned in similar reports [11, 54, 55]. Laws and policies that are discriminatory and punitive are also believed to be responsible for pushing this community away from health care services and undermining public health efforts [9, 11]. These factors, along with a lack of trust in the health care system, were echoed in the results of similar studies involving transgender women from within the country [2, 11, 55] and other parts of the globe [44–47]. Lack of gender affirming care with minimal training provided to doctors and staff in managing trans-specific health concerns has been repeatedly stated in studies from Malaysia [2, 12, 25, 40, 56]. Transgender women in the current study felt repeatedly targeted as a “key population”, especially in HIV-related health promotion activities, often being grouped with MSMs. This paradox of “health campaign fatigue”, in the authors’ opinion, has much to say about the accessibility of health care to urban transgender women in Malaysia, much closely resembling experiences by transgender women in more developed parts of the world [57]. However, being subsumed in health campaigns with MSMs has been reported to negatively impact transgender women’s uptake of HIV care due to inadequate representation [2]. 

### Sexual practices relating to oral transmission of STIs

The barriers and enablers of safe sexual practices, particularly in relation to oral transmission of STIs, were also explored. Similar to findings from recent reports, the participants in the study were well aware of HIV transmission and the importance of condoms for safe sex, both genital and anal sex [58, 59]. Oral sexual practices involving receptive fellatio and anilingus for clients or partners were common [10]. However, transmitting STIs was not particularly perceived as a risk through the oral route. Gaps in knowledge regarding oral STIs and risks associated with oral sex were similar to the gaps in knowledge reported even in other gender-diverse populations as well as heterosexual populations [16, 19, 39, 60]. Several factors contributing to unprotected oral sex, including low perception of risk, fear of losing sex work clients, misconceptions about safe practices, and concerns about compromising with a high-paying client, were similar to those cited in global literature [16, 39, 61]. The traditional focus of public health campaigns on condom use for anal and genital sex may contribute to an underestimation of the risk of STI transmission through oral sex [60, 62]. Even though the knowledge of the spread of infection may have been heightened in general owing to the recent pandemic, the spread of infection via the oral route was not particularly seen as a concern. The risk of transmission of HIV through saliva and healthy oral mucosa may be none or negligible [17], but the same is not true for other viral or bacterial STIs [16]. With growing concerns of antibacterial resistance with STIs like N. gonorrhoea [17], it is recommended that awareness regarding the transmission of STIs via oral sexual practice be more openly discussed and more widely incorporated into public health interventions [63]. 

Meanwhile, Human Papillomavirus (HPV) transmission via oral sex and the development of oral and oropharyngeal cancers also need emphasis in both key populations and the lay public [19]. Hence the emphasis is on the wider need for public health campaigns with comprehensive sexual education that includes oral sex as a transmission route for STIs. While traditional STI prevention efforts have focused on genital transmission, the rise of antibiotic-resistant infections and HPV-related oral cancers has led to a call for broader discussions about oral sexual practices [16, 64]. This is more relevant for vulnerable populations such as transgender women.

This unique qualitative exploratory research combines the study of oral health care needs, the utilisation of dental services, and the oral transmission of STIs. It highlights the critical role of oral health care providers in the early detection of oral STIs, particularly in light of rising global STI rates [65], increasing antibiotic resistance [63] and the growing incidence of HPV-related oral cancers [66]. The valuable insights gained through this research will be used to develop a tailor-made health promotion tool to benefit local transgender women and similar vulnerable communities.

### Strengths and limitations of this study

The strength of this study lies in its contribution to the limited body of research on the oral health needs and dental service utilisation of transgender women in resource-limited countries. Additionally, the use of qualitative analysis helped to explore the unique social and interpersonal challenges that contribute to the unmet healthcare needs of this vulnerable and marginalised population. Furthermore, the active involvement of transgender women in study design, participant recruitment and feedback on preliminary data interpretation enhanced the study’s inclusivity and relevance.

A key limitation of this study is that the findings reflect the perceptions and experiences of a small group of urban transgender women, which may not fully capture the perspectives of those in rural settings. A larger, more diverse sample could provide broader insights with varied inferences. Additionally, as this was a preliminary exploratory study, biological data, including biochemical, virological, and microbiological (STI screening) data, were not collected. Instead, self-reported information on HIV/STI testing was recorded, which may be subject to recall or reporting biases.

## Conclusion

Our findings from this qualitative analysis provide a perspective of a small group of transgender women living in an urban part of northern Malaysia and reflect the sentiment within the community. The findings from the study provided a nuanced understanding of the community’s needs. Although aesthetics was prioritised, a general lack of awareness regarding the importance of oral health was noticed, along with poor dental service utilisation. Gaps in knowledge of oral STIs and perceived low risk of transmission of STIs through oral sex were reported. The research underscores the importance of regular oral health examinations as a preventive measure in addressing these public health concerns. As healthcare providers who routinely examine the oral cavity, dentists may be able to offer valuable education on the risks of oral sex and the importance of regular screenings for sexually active individuals. The research underscores the importance of incorporating the risks associated with oral transmission of STIs into comprehensive sexual health promotion efforts by healthcare professionals, researchers, and other stakeholders in public health education.

## Electronic supplementary material

Below is the link to the electronic supplementary material.

Supplementary Material 1: Guide to in-depth interviews.

Supplementary Material 2: GRIPP2-SF checklist.

Supplementary Material 3: Standards for Reporting Qualitative Research (SRQR) checklist.

## Data Availability

Due to the sensitive nature of the data collected in this study, it cannot be made publicly available. The data involves personal and confidential information, and sharing it could compromise participant privacy. However, specific aspects of the data may be made available upon reasonable request to the corresponding author, subject to approval by the ethics committee and in compliance with data protection regulations.
