# Supplementary material for: Exploring the barriers and enablers of oral health care utilisation and safe oral sex practices among transgender women in Malaysia: a qualitative study
Source: BMC Public Health. 2025 Apr 3;25:1261. doi: 10.1186/s12889-025-22417-9 (PMC11969983; doi:10.1186/s12889-025-22417-9)
Supplement: Supplementary file 1 — Supplementary Material 1: Guide to in-depth interviews. [file 12889_2025_22417_MOESM1_ESM.docx]

**Guide to Semi-structured In-depth Interviews**

| Research Objectives | Broad Themes- Deductive Approach | Interview Questions |
| --- | --- | --- |
| 1. Explore oral health care needs 2. Understand requirements for dental health care service utilization 3. Identify barriers and enablers to safe sexual practices particularly relating to oral transmission of STIs | Place in the community and social support | - Tell me about your transition journey - Tell me about your relationships with family, friends, and partners. - How did you cope with transition related issues? |
|  | Oral health care | - What is your awareness of dental health? - Do you think that oral health is important for transgender women? |
|  | Visiting the dentist | - Tell me about your experience at the last dental visit - How can we motivate transgender women to visit dentists? |
|  | Sexual health and practices | - Tell me about your sexual practices - Tell me about your awareness of HIV/STIs - How do you use protection against HIV/STIs |
|  | Oral sex and protection | - Do you think transmission of infections can occur with oral sex? - Do you think it is important to use protective measures during oral sex? - Has oral sex been discussed as a source of STI transmission by your health care provider? |
