# Supplementary material for: Exploring the barriers and enablers of oral health care utilisation and safe oral sex practices among transgender women in Malaysia: a qualitative study
Source: BMC Public Health. 2025 Apr 3;25:1261. doi: 10.1186/s12889-025-22417-9 (PMC11969983; doi:10.1186/s12889-025-22417-9)
Supplement: Supplementary file 2 — Supplementary Material 2: GRIPP2-SF checklist. [file 12889_2025_22417_MOESM2_ESM.docx]

**Guidance for Reporting Involvement of Patients and the Public (GRIPP-2) Short form Checklist**

| **Section and topic** | **Item** | **Reported on page No** |
| --- | --- | --- |
| 1. Aim | The aim of PPIE in this research was community engagement. Transgender women were involved in the study's design and recruiting participants. They also provided feedback on the preliminary data interpretation. | 2,4 |
| 1. Methods | A participatory approach for designing the study was adopted, with transgender women from the local community involved in planning. Initial meetings between the principal author (LAT) and a few transgender women community advocates were conducted in informal settings to explore the general frame of mind of the community. Community advocates provided feedback regarding the study design and assisted with recruiting participants. They not only supported the interpretation of the preliminary data but also the follow-up part of the study.  The initial participants were referred by a local community-based welfare organisation working with transgender women. Subsequent participants were recruited through word of mouth using a snow-balling sampling method and belonged to several friendship networks.  The interview guide was developed by the authors based on prior research evidence in local transgender health (6, 35) and was guided by community advocates who were involved in healthcare outreach.  They were conducted by both the principal author (LAT), a cis-gender woman, and a trained research assistant (HSD), who is a transgender woman with experience in transgender health outreach and advocacy. English and Bahasa Malaysia were both used during the interviews, with facilitation by the research assistant to better understand variations in local dialects and appropriate cultural nuances. | 6- 8 |
| 1. Study results | Positive outcomes of PPI in the research project-   - Exploratory research with experienced persons from transgender community - Recruitment of participants - Culturally appropriate language usage in the interviews - Data interpretation with PPI involvement   Limitation of outcome of PPI in the research project-   - Time taking - Engagement with only those who are within the closed network - Payment or reimbursement | 6-14 |
| 1. Discussion and conclusions | Since this project was exploratory in nature and involved a marginalised population, PPI was a crucial element in ensuring that the participants felt comfortable and safe whilst narrating their lived experiences. | 21-26 |
| 1. Reflections /critical perspective | The project greatly benefited from the involvement of Patient and Public Involvement (PPI), particularly in the area of recruitment. Building trust is crucial when working with marginalised populations, and the contributions of community members, especially those from the Penang Family Health Development Association (FHDA), were instrumental in engaging these groups. The expertise of Malaysian transgender women was invaluable in understanding cultural nuances and interpreting qualitative data effectively. | 9,25,26 |
